# Clinicopathological and molecular studies on cattle naturally infected with lumpy skin diseases in selected districts of Wolaita Zone, Southern Ethiopia

**DOI:** 10.1186/s12917-022-03403-4

**Published:** 2022-08-03

**Authors:** Mesfin Mathewos, Fistum Dulo, Zewdneh Tanga, Melaku Sombo

**Affiliations:** 1 School of Veterinary Medicine, Wolaita Sodo University, Wolaita Sodo, Ethiopia; 2 National Animal Health Investigation and Diagnostic Center, Sebeta, Ethiopia

**Keywords:** Cattle, Histopathology, “Lumpy skin disease virus (LSDV)”, Real-time polymerase chain reaction, Wolaita zone

## Abstract

**Background:**

Lumpy skin disease is a contagious viral disease of cattle caused by LSDV that results in huge economic losses in the cattle industry. This study characterizes LSDV in cattle through clinicopathological and molecular techniques in selected districts of Wolaita Zone, Southern Ethiopia.

**Methods:**

A crossectional study was conducted from November 2020 to June 2021 using Real-time polymerase chain reaction and Histopathological techniques to confirm LSDV.

**Result:**

This study revealed that the percentage of positivity of cattle for LSDV was 36.2%. Clinically, cattle infected with LSDV revealed fever (39–41 °C), nodular lesions on the skin and mucous membranes, and lymphadenopathy. Histopathologically, affected tissue revealed ballooning degenerations of the epidermis, infiltration of mononuclear inflammatory cells, vasculitis, and intracytoplasmic eosinophilic inclusion bodies. RT-PCR confirmed that DNA extracts from skin biopsies of virus isolates were positive for LSDV.

**Conclusion:**

The present study confirms that LSDV is widely circulating in cattle of selected districts of the Wolaita zone. Thus, effective control measures through regular vaccination and further confirmation of circulating strains of LSDV through detailed molecular analysis should be recommended.

## Introduction

Poxviruses are complex, linear, enveloped, double-stranded DNA viruses with large genomes [48], which are responsible for several economically significant zoonotic diseases affecting humans, wildlife, farm animals, and domestic animals [23]. They belong to the Poxviridae family, which is divided into two subfamilies: Entomopoxvirinae, which infect invertebrates, and Chordopoxvirinae, which infect vertebrates [30, 42, 45]. Among the *Chord poxvirus* subfamily, *Capripoxvirus* is capable of infecting the cattle, sheep, and goats, which is comprised of goat pox virus (GTPV), sheep pox virus (SPPV), and LSDV and *Parapoxvirus* [5, 16, 21, 48, 72].

Lumpy skin disease (LSD) is a highly contagious, fatal skin disease of cattle and water buffalos caused by LSDV, a member of the Poxviridae family [53], and results in important socioeconomic transboundary infection [42, 72]. The disease is designated as "LSD," "Pseudo-urticaria," "Neethling viral sickness," "exanthema nodularis bovis," and "knopvelsiekte" [8, 27, 40, 70, 71]. Historically, LSDV was first documented as an epidemic in Zambia in 1929 [51, 63] and since then has spread out of Africa into the Middle East region, southern Russian Federation, Central Asia, Western Europe, and Central-Eastern Europe [50, 70, 71].

Lumpy skin disease causes significant economic losses due to persistent debility, slowed development, decreased milk and meat output, hide damage, and sterility in bulls, infertility, abortion, and different ranges of mortality and morbidity [29, 42, 43]. The infection rate has been 1 to 2% but in some areas, it may reach 80 to 90%. The mortality rate has been reported about 10–40%, and even higher in special cases, but the usual rate was 1 to 5% [25, 64, 65].

To date, the most likely vectors for LSDV transmission are blood-sucking arthropods such as stable flies (*Stomoxys calcitrans*), mosquitoes (*Aedes aegypti*), and hard ticks (Rhipicephalus and Amblyomma species). New evidence suggests that the *Musca domestica*, may also play a role in LSDV transmission, but this has not yet been tested in a clinical setting [64, 73]. Fever, nodular development, the fast eruption of skin nodules, enlarged superficial lymph nodes, generalized lymphadenitis, and edema are all symptoms of LSDV [4, 57, 62].

The tentative diagnosis of LSD is mainly depending on the typical clinical signs and postmortem examination. Although a combination of histopathological techniques with electron microscopy, serology, molecular assays, and viral isolation can provide a definitive detection for LSDV [44, 70, 71, 79]. The molecular detection of LSDV using *Capri poxvirus*-specific primers for the attachment protein and fusion protein gene using conventional and Rt-PCR techniques have been documented to be used on blood, tissue, and semen specimens [2–4].

Lumpy skin disease was first observed in the southwest of Lake Tana in 1983 [46], but now it has been spread to almost all the regions and agro-ecological zones of the country. LSD has become one of the most economically important livestock diseases in Ethiopia that results in hide damage, prolonged loss of productivity of dairy and beef cattle, weight loss, abortion, infertility, and sometimes permanent sterility, and denied access to both local and international markets. Consequently, information regarding Clinico-pathological and Molecular studies was limited and no studies were done on cattle naturally infected with LSDV in selected districts of the Wolaita zone. Therefore, the objective of this study was to characterize LSDV in cattle through clinicopathological and molecular techniques in selected districts of Wolaita Zone, Southern Ethiopia.

## Material and methods

### Study area

The study was conducted in a selected district of the Wolaita zone namely Humbo and Sodo town, southern Ethiopia. Wolaita Sodo is located about 390 km south of Addis Ababa. The area is located at the latitude of 8°50°N and a longitude of 37°45°E. Topographically, the area is marked by hilly, flat, steep slopes and gorges and several streams and mountains. The highest mountain is Damota, 2500 m.a.s.l, which is located near Sodo town. The altitude varies from 1100–2950 m.a.s.l. The area experiences a mean annual temperature of about 20 °C. The mean maximum temperature is 26.2 °C and the average monthly minimum temperature is 11.4 °C. The rainfall regimes over much of the area are typically bimodal with the small rainy season occurring from February to April and a big rainy season extending from June to September. The mean annual rainfall of the area ranges from 450–1446 mm with the lowest being on low land and the highest on high land (WZFSD. Report on Food Security Activities Presented to Zonal Council; 2013). The livestock population in the study area is estimated to be 2,982,513 cattle, 1,285,161 sheep, 1,599,081 goats, 23,412 horses, 20,283 mules, 330,214 donkeys, and 3,116,356 chickens [76].

### Study population

Those cattle infected with LSDV irrespective of their age, sex, breed, body condition, and farming system were included in this study.

### Study design and sampling technique

A cross-sectional study was conducted on cattle suspected for LSDV from November 2020 to June 2021 to characterize LSDV in cattle through clinicopathological and molecular techniques in selected districts of Wolaita Zone, Southern Ethiopia. The animal that showed high fever between 39 to 41 °C, visible skin nodules, enlarged lymph nodes, and lacrimation were selected and investigated through different diagnostic approaches for identification and characterization of circulating LSDV in the study area.

### Sample collection and anesthetic procedures

The representative samples for molecular characterization were collected from clinically sick animals according to the procedures [52]. Before sampling the animal was restrained and then a detailed physical examination was done on sick animals. Once these procedures were accomplished, the sampling area was disinfected with alcohol, and the hairs were removed with the help of a sterile scalpel [24].

#### Anesthetic procedures and the anesthetic agent used

An 18-gauge 3.8-cm needle was directed perpendicular to the skin surface. Once the skin was penetrated, place a drop of local anesthetic solution in the hub of the needle. The needle should then be advanced slowly until the anesthetic solution was drawn into the subcutis. The anesthesia agent that was used is Lidocaine. Approximately 2% Lidocaine HCl (0.2 mg/kg body weight) was infiltrated into the subcutis before sampling as previously described by [12].

Then after, the incision of the nodule was done using a sterile surgical scalpel blade by holding the tissue with tissue forceps. A total of 20 biopsy samples were taken aseptically (Table 1) and then placed immediately into the universal bottle containing 10% neutral buffered formalin until the tissue sample was processed at Hawassa University faculty of veterinary pathology. Moreover, for molecular diagnosis samples were collected using normal saline water and which was transported by icebox to NAHDIC molecular laboratory and kept at + 4 °C until genomic DNA extraction as described by [6, 51]. Following incision, wound spray was used to prevent the contamination of the wound by flies.

**Table 1:** List of collected samples from the study areas

Sample No	Origin	Sample type	Fixative used		Type of Dx used	
			10% neutral buffered formalin	NaCL	Histopathology	Molecular
LSDH1	Humbo	Skin nodule(Tissue)	√		√	
LSDH2	Humbo	Skin nodule(Tissue)	√		√	
LSDH3	Humbo	Skin nodule(Tissue)	√		√	
LSDH4	Humbo	Skin nodule(Tissue)	√		√	
LSDH5	Humbo	Skin nodule(Tissue)	√		√	
LSDH6	Humbo	Skin nodule(Tissue)	√		√	
LSDH7	Humbo	Skin nodule(Tissue)	√		√	
LSDH8	Sodo town	Skin nodule(Tissue)	√		√	
LSDH9	Sodo town	Skin nodule(Tissue)	√		√	
LSDH10	Sodo town	Skin nodule(Tissue)	√		√	
LSDH11	Sodo town	Skin nodule(Tissue)	√		√	
LSDH12	Sodo town	Skin nodule(Tissue)	√		√	
LSDH13	Sodo town	Skin nodule(Tissue)	√		√	
LSDP21	Sodo town	Skin nodule(Tissue)		√		√
LSDP22	Sodo town	Skin nodule(Tissue)		√		√
LSDP23	Sodo town	Skin nodule(Tissue)		√		√
LSDP24	Sodo town	Skin nodule(Tissue)		√		√
LSDP25	Sodo town	Skin nodule(Tissue)		√		√
LSDP26	Sodo town	Skin nodule(Tissue)		√		√
LSDP27	Sodo town	Skin nodule(Tissue)		√		√

### Clinical and laboratory diagnosis

#### Clinical examinations

During the study period, all suspected cases from Humbo district and Sodo town were clinically examined for the presence of skin nodules on the head and neck region, perineum, genitalia, udder, limbs, and as well as for other clinical signs using appropriate restraining techniques as previously described by [17, 38].

#### Histopathological examination

A biopsied skin tissue sample was fixed in buffered formalin and then processed in an automatic tissue processor and embedded in paraffin blocks and sectioned at 5 μm thickness. Sections were then stained with Haematoxylin and Eosin (H&E) according to previous methods described by [66], and the images were acquired with Olympus digital microscope to examine the histopathological changes.

#### DNA extraction

DNA was extracted by Qiagen kit, according to the manufacturer’s instructions as described by [55].

### Polymerase chain reaction

The test was carried out according to the protocol followed by [74]. LSDV DNA amplification was done using by Qiagen kit, according to the manufacturer’s instructions. Real-time polymerase chain reaction (RT- PCR) assay was used to detect the LSDV using Eva Green supermix 10 µl with following primers 2 µl each, forward primer:-CP-HRMSb-Fow-5 pm/µl 5’GGTGTAGTACGTATAAGATTATCGTATAGAAACAAGCCTTTA-3’ and reverse primer CP-HRM l1REV-5 pm/ µl 5’-AATTTCTTTCTCTGTTCCATTTG-3’and DNA template 3 µl was used for amplification of PCR. After amplification of the DNA template, the positive samples were noted by amplification fluorescence curves, melting curves, and cycle threshold (Ct) values from the assay which were used to describe the positive samples: Ct values with no or higher than 37 were indicated as negatives suggesting the absence of the virus from the tissue specimens. The optimized cycle program for RT-PCR consisted of the following thermal cycles conditions were used: an initial denaturation step at 95 °C for 3 min, followed by 40cycles in three steps: denaturation at 95 °C for 15 s, annealing at 58 °C for 1.20 s, and elongation at 72 °C for the 30 s with the fluorescence recording at the end of the combined annealing elongation step.

### Statistical analysis

The collected data were recorded on Microsoft Excel spreadsheets. Descriptive statistics like percentage was used to calculate positivity by dividing the number of LSD positive animals by the total number of animals tested. Finally, all data were analyzed by using Stata software version 13.

### Ethics approval and consent to participate

The study was carried out in compliance with the ARRIVE guidelines. This research project was approved by the School of Veterinary Medicine-Animal Care and Ethics committee of Wolaita sodo university (Protocol No. WSU 41/25/1292). Tissue samples were collected during routine veterinary practice in adherence to a high standard of veterinary care, and after the permission of the dairy farms’ owners. During the study, all methods were performed following the relevant guidelines and regulations.

## Results

### Clinical and gross lesion characterization

**Fig. 1:**
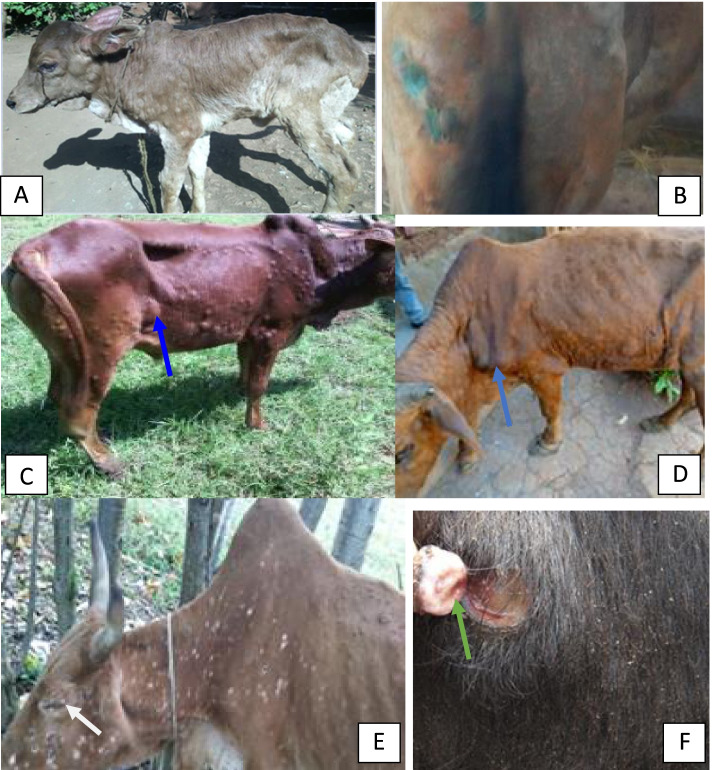
Clinical signs and gross lesions of cattle affected by LSD. Distribution of skin nodules through the body surface of a calf (**A**), swelling of udder accompanied by mastitis (**B**), enlargement of prefemoral (**C**), and prescapular lymph node (**D**), corneal opacity or keratitis (**E**), and sit fast lesion (**F**)

Clinical examination of cattle suspected for LSDV (384) revealed the percentage of positivity of cattle for LSD was (139/384) 36.2%. Clinically, LSD was characterized by fever (39–41 °C), nodular lesions on the skin and mucous membranes, and lymphadenopathy. Although, other clinical signs such as lameness, dyspnea, nasal discharge, rough hair coat, mastitis, corneal opacity, severe debilitation, anorexia, depression, lacrimation, and salivation have been noted during the outbreak investigation (Fig. 1).

### Histopathology

**Fig. 2:**
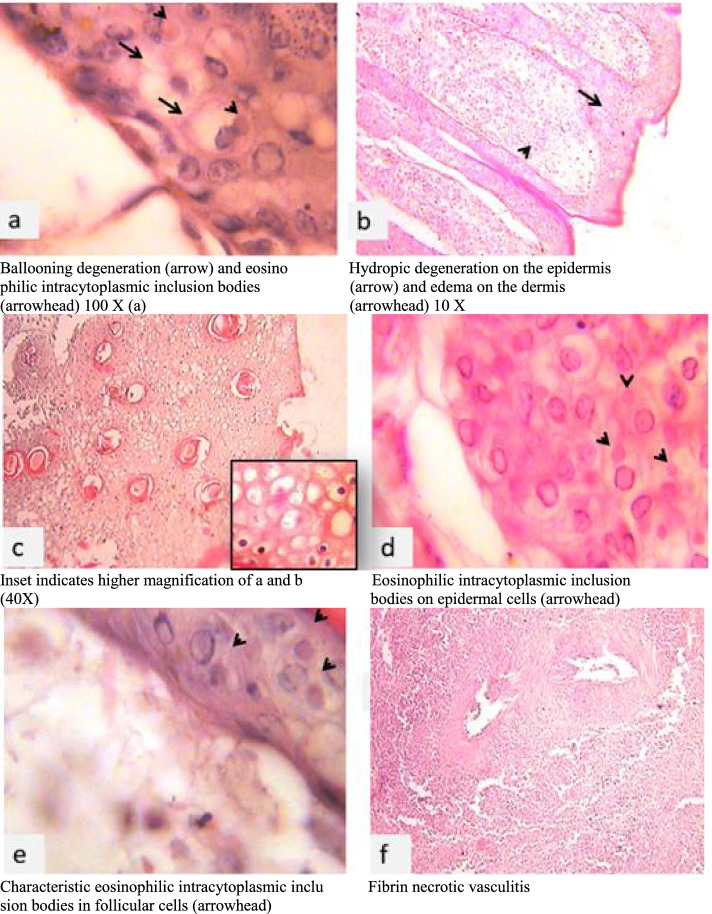
Histopathology of LSD: H&E.Ballooning degeneration (arrow) and  intracytoplasmic eosinophilic inclusion bodies (arrowhead) 100 X (**a**). Hydropic degeneration on the epidermis (arrow) and edema on the dermis (arrowhead) 10 X (**b**). Inset indicates higher magnification of a and b (40X) (**c**); Eosinophilic intracytoplasmic inclusion bodies on epidermal cells (arrowhead) (**d**). Characteristic eosinophilic intracytoplasmic inclusion bodies in follicular cells (arrowhead) (**e**); Fibrin necrotic vasculitis (**f**)

Histopathologically, this study revealed a hydropic (ballooning) degeneration of keratinocytes, eosinophilic intracytoplasmic inclusion bodies on epidermal cells and follicular cells, and disrupted blood vessel wall by infiltration of mononuclear inflammatory cells and fibrin deposits (Fig. 2).

### Molecular detection

As indicated in Table 2, all samples that have a Ct value lying between (14.82 and 23.25) were positive. No or higher values were indicated as negatives in which lower or no loads of the virus are present.

**Table 2:** Real-time PCR Ct values of LSD suspected tissue samples

S/r No	sample code	Ct value	Result
1	LSDP21	16.06	Positive
2	LSDP22	17.13	Positive
3	LSDP23	20.77	Positive
4	LSDP24	22.85	Positive
5	LSDP25	23.25	Positive
6	LSDP26	18.49	Positive
7	LSDP27	14.82	Positive
8	SPPv + ve control	32.67	Positive
9	GTPV + ve control	32.30	Positive
10	LSDV + ve control	26.52	Positive
11	Negative control	Undetected	Negative

**Fig. 3:**
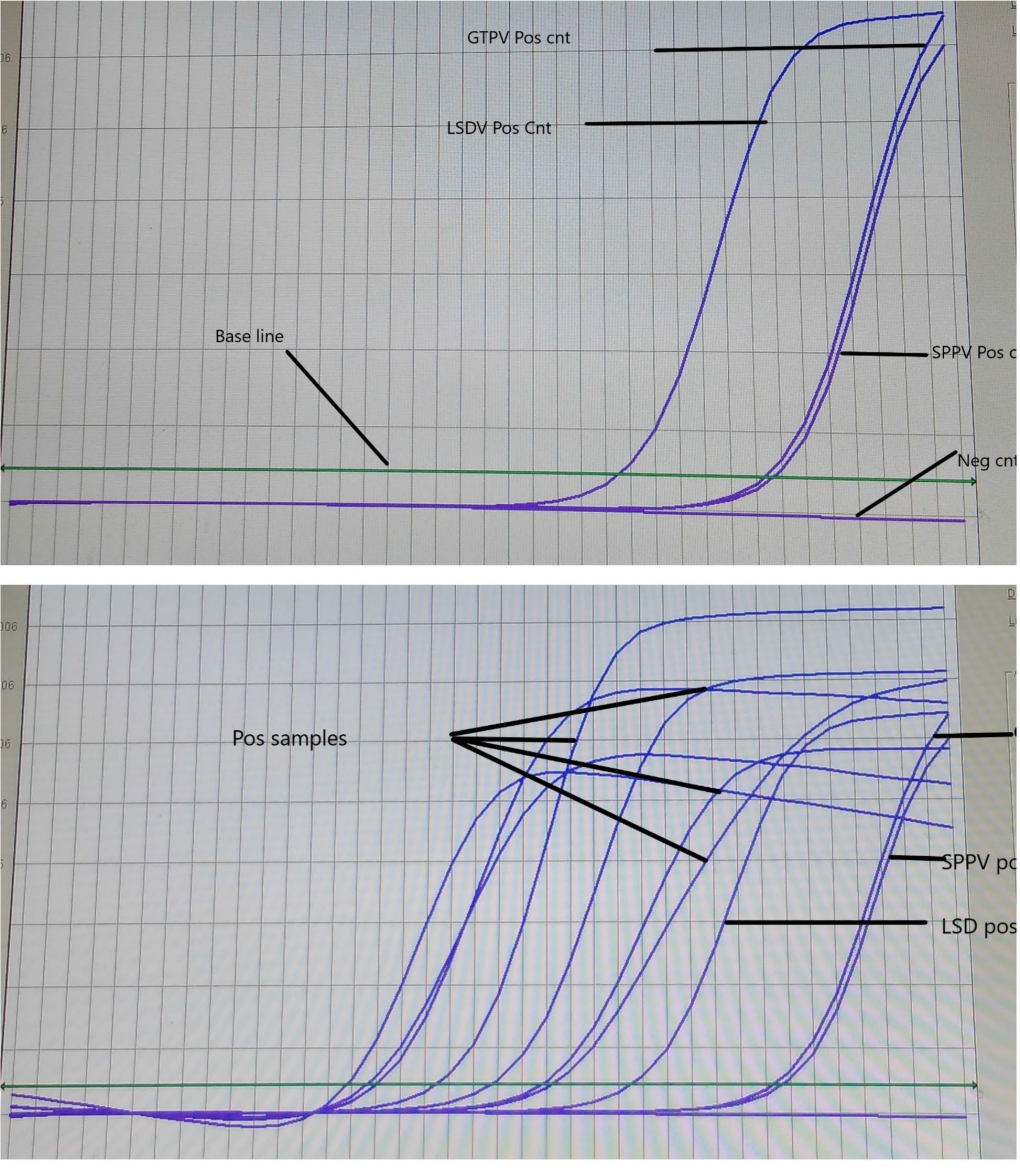
Amplification fluorescence curves

PCR proved to be the best choice for prompt detection of LSDV outbreaks. All the 7 extracted DNA samples from skin nodules amplified by real-time PCR were positive for LSDV. None of the negative controls produced any amplicons. Below are indicated amplification curves of the real-time PCR (Fig. 3).

## Discussion

Lumpy skin disease was restricted to Africa wherein it led to several devastating pandemics in several countries including Ethiopia, thereby threatening food security and consequently increasing poverty [34, 58, 64]. In the present study, LSD was investigated in two selected districts of Wolaita Zone (Humbo and Sodo town), southern Ethiopia. The percentage of positivity of cattle for LSDV was 36.2%.This percentage of positivity was higher than the previous report described by [10, 15, 19, 35, 41, 47, 49] and [69] who recorded 21.2%, 18%, 15.71%, 13.61%, 8.77%, 7.4%, 6.1%, and 5.69% from Ethiopia, Bale zone, South Wollo zone, central Ethiopia, central Ethiopia (Asela, Bishoftu, Akaki, and Holeta Genet towns), north-eastern Ethiopia, Borena Zone, and East Hararghe and East Shewa zone, respectively. Other authors reported a wide range of percentage of positivity ranging from 0.65 up to 85% [9, 18, 26, 36, 68, 72] from Iraq, Great Britain, Africa, the Near East, Turkey, Greece, and the Middle East and Asian countries, respectively. The genetic difference, immunity status, geographic location, climate, and virulence of virus strain were raised for percentage of positivity variation [4, 26].

Clinically, LSD was characterized by fever (39–41 °C), nodular lesions on the skin and mucous membranes, and lymphadenopathy. This finding was in agreement with the previous report described by [56, 69]. Although, other clinical signs such as lameness, dyspnea, nasal discharge, rough hair coat, mastitis, corneal opacity, severe debilitation, anorexia, depression, lacrimation, and salivation have been noted during the outbreak investigation. Similarly, [7, 14, 19, 20, 25, 28, 31–33, 39, 59, 77, 78] have recorded the same symptoms in natural and experimental infections. Grossly, all affected cattle have resembled circumscribed nodules with different sizes on the skin covering all over the body surface such as the head, neck, trunk, perineum, udder, and teats. The surface of the nodule was reddish-gray and edematous in the sub-cutis layer upon incision of the nodules. In many infected animals, the necrotic nodules were ulcerated and formed deep scabs (sit fast). Similar reports were previously described by [6, 16, 59, 75].

Histopathologically, this study revealed there was a hydropic (ballooning) degeneration of keratinocytes, disorientation of striated muscle striations, edematous dermis, eosinophilic intracytoplasmic inclusion bodies on epidermal cells and follicular cells, and disrupted blood vessel wall by infiltration of mononuclear inflammatory cells and fibrin (fibrin necrotic vasculitis) deposits. These histopathological findings were previously reported by [1, 11, 22, 25, 54, 61, 67, 73].

Molecular detection of samples suspected of LSDV has shown Ct values between (14.82 and 23.25). This was in agreement with another finding which reported Ct values less than 37 as positives [60]. PCR proved to be the best choice for prompt detection of LSDV outbreaks, especially other methods that are believed to be time-consuming [28]. A range of real-time PCR assays [13] was used in diagnostic laboratories. All the 7 extracted DNA samples from skin nodules amplified by real-time PCR were positive for LSDV. This was in agreement with several studies [28, 37] in which the virus in skin lesions was reported with a level of success of 100%.

## Conclusion and recommendations

This was the first study reporting on the Clinicopathological and molecular studies of LSDV in naturally infected cattle in humbo districts and Sodo town. The presence of characteristic LSD clinical features, RT-PCR results, and the presence of characteristic tissue effects on histopathological examination indicated that the lumpy skin disease virus is widely circulating in cattle in the study area. Thus, a detailed molecular analysis of an isolate within the study district needs to be carried out to produce a strain-specific vaccine to maintain the well-being of the animal and enhance its production and productivity.

## Data Availability

The datasets used and analyzed during the current study are available from the corresponding author on reasonable request.

## Abbreviations

CaPV: Capri pox virus
Ct: Cycle threshold
dsDNA: Double-stranded deoxyribonucleic acid
EDTA: Ethylene diamine tetraacetic acid
GTP: Goat pox
GTPV: Goat poxvirus
LSD: Lumpy skin disease
LSDV: Lumpy skin disease virus
NAHDIC: National animal health diagnostic and investigation center
OIE: Office International des Epizooties
PCR: Polymerase Chain Reaction,
RT-PCR: Real-Time Polymerase Chain Reaction
WZFSD: Wolaitta Zone Food Security Department
